# Clinical practice of childhood apraxia of speech in Hong Kong: A web-based survey study

**DOI:** 10.1371/journal.pone.0284109

**Published:** 2023-04-07

**Authors:** Eddy C. H. Wong, Min N. Wong, Shelley L. Velleman

**Affiliations:** 1 The Department of Chinese and Bilingual Studies, Faculty of Humanities, The Hong Kong Polytechnic University, Hung Hom, Hong Kong SAR, China; 2 Research Centre for Language, Cognition, and Neuroscience, the Hong Kong Polytechnic University, Hong Kong SAR, China; 3 Research Institute for Smart Ageing, the Hong Kong Polytechnic University, Hong Kong SAR, China; 4 Department of Communication Sciences and Disorders, the University of Vermont, Burlington, Vermont, United States of America; Kasturba Medical College Mangalore / Manipal Academy of Higher Education, INDIA

## Abstract

**Background:**

A survey study on clinical practice not only provides insight into the implementation of knowledge, but also informs future investigations. There is a limited understanding of childhood apraxia of speech (CAS) in Cantonese speakers. This study examined the clinical practice of CAS in Hong Kong and discussed future directions of research for better evidence-based practice.

**Methods:**

Qualified Hong Kong pediatric speech-language pathologists (SLPs) completed the online questionnaire, which had a total of 48 questions regarding their knowledge of and experience with CAS in Cantonese speakers, including assessment, diagnosis, and treatment.

**Results:**

Seventy-seven responses were received from Hong Kong SLPs. Most of the SLPs (83.2%) rated their understanding of CAS as either “a little” or “fair”. About half (53.2%) of the respondents had worked with children with CAS. No standardized assessment or objective/quantitative measures were used clinically. Instead, seven assessment tasks, including imitation of polysyllabic words and speech and language samples were used commonly. Perceptual judgment of clinical features is still the most popular approach for diagnosis, with a variety of lists in use. Of concern was that, in addition to using some evidence-based approaches, local SLPs treated CAS using approaches that have limited evidence, in the context of less treatment frequency, targeting both speech and language skills within the same session, and with only partial implementation of the approaches.

**Conclusions:**

The results suggest that the understanding of CAS among local SLPs requires attention. One reason for this is that evidence regarding the assessment, diagnosis, and treatment of Cantonese speakers with CAS is still limited. Future investigations are needed.

## Introduction

Childhood apraxia of speech (CAS) is a type of motor speech disorder characterized by impairment of motor planning and/or programming of speech movements [1]. The estimated population-based prevalence of CAS is one in 1,000 children [1]. Clinical features, assessment methods, diagnostic standards, and treatment approaches have been studied empirically in the past decades in English speakers with CAS [2–4].

The core impairment of CAS manifests a variety of clinical features. Three consensus features have been identified, including token-to-token inconsistency, deficits in co-articulatory transitions between sounds and syllables, and lexical stress errors [2]. Other clinical features include vowel and consonant errors, within-speech and non-speech groping, voicing errors, intrusive schwa, slow speech rate, and increased difficulty with multisyllabic words [5].

Due to the lack of clinical marker(s) that have empirical diagnostic accuracy for CAS [6], assessment and diagnosis of CAS in children are challenging. Fortunately, tests or tasks such as the Dynamic Evaluation of Motor Speech Skill (DEMSS) [7], maximum performance tasks (MPT) [8], and syllable repetition tasks (SRT) [9] provide information about motor speech skills in children, aiding the differential diagnosis of CAS. Nevertheless, CAS diagnoses are often still based on expert perceptual judgments of clinical features [5]. In response to this method, checklists that aim to assist with CAS diagnosis have been proposed. Strand’s 10-point checklist [10] is one of the popular checklists used in research and clinical practice. Some of the features in the checklist had 91% diagnostic accuracy in one study, namely syllable segregation, lexical stress errors, articulatory accuracy on polysyllabic words, and repetition of [pǝtǝkǝ] [4]. With these available tools as guidelines, a recent survey study found that about 60% of SLPs respondents had moderate or high levels of confidence in their ability to make a differential diagnosis of CAS [11].

Once a CAS diagnosis is confirmed, treatment should be provided. There are numerous treatment approaches that have been empirically studied. The Nuffield Dyspraxia Programme (NDP) [12], Rapid Syllable Transition treatment (ReST) [13], Dynamic Temporal and Tactile Cueing (DTTC) therapy [14], and Prompts for Restructuring Oral Muscular Phonetic Targets (PROMPT) [15] are evidence-based treatments for children with CAS. These treatments target both segmental accuracy and the prosodic skills of children with CAS. NDP and ReST have been investigated in randomized control trials with promising outcomes [16], while DTTC and PROMPT have been reported based on controlled studies [15, 17, 18]. Other treatment approaches with emerging evidence include ultrasound biofeedback [19], integrated phonological awareness intervention [20], augmentative and alternative communication [21], the combined treatment of the stimulability approach and the modified core vocabulary approach [22], the Speech Motor Learning approach [23], the Syllable Repetition Method [24], Melodic Intonation Therapy [25], and others. Some other commercially available treatment programs, such as the Kaufman Speech to Language Protocol [26] and Talktool® Oral Placement Therapy [27], have limited to no published evidence [28].

### CAS in other languages

The existing understanding of CAS is mainly based on English and Dutch speakers, but the literature about CAS in children who speak other languages is growing. For example, the genetic and neural bases of CAS have been investigated in Chinese [29] and Italian [30]; clinical features or speech performance have been explored in Arabic [31], Cantonese [32, 33], Mandarin [34], and French [35]; assessment tests or diagnostic checklists have been developed for Arabic [36], Brazilian Portuguese [37], Danish [38], and Swedish speakers with CAS [39]; and the treatment efficacy of different approaches has been examined in speakers of Brazilian Portuguese [40, 41], Cantonese [42], German [43], Italian [44], Korean [45], Mandarin [46], Spanish [47], and Turkish [48].

### Importance of survey studies

Apart from research practice, researchers are always interested to know trends in clinical practice, especially whether research information has been well received by clinicians and what is in need of further investigation. Obtaining information from clinicians using surveys is one direct method to achieve these goals. Information about clinical practice with English speakers who have CAS has been obtained from SLPs via survey with respect to diagnostic criteria [49] and clinical management [50, 51], as well as the development of expertise [52]. These survey studies have provided the basis for further investigation of specific topics. For example, the identification of more than 50 clinical features used by SLPs early in the millennium [49] highlighted the need for diagnostic markers and criteria, resulting in the proposal of three consensus features by the American Speech-Language-Hearing Association (ASHA) [2]. Another study found that SLPs in Australia and New Zealand use NDP as their primary intervention approach, but most of them only use it as one component of their intervention for children with CAS [51]. The authors reported that SLPs in the US mostly use the Kaufman Speech to Language protocol [26] and DTTC, while SLPs in Canada mostly use PROMPT. Again, these approaches were used as one component of overall intervention for children with CAS. This underscores the need for investigation of the effectiveness of available evidence-based treatment programs in real clinical settings. Randazzo [52] suggested that clinicians develop their CAS expertise mainly based on continuing education programs. This drew attention to the content of these profit-making courses.

Survey studies in some non-English speaking countries have played an initial role in exploring CAS in those populations. The clinical features of CAS in Swedish-speaking children were explored [53]. The results showed that the clinical manifestations of CAS in Swedish speakers are similar to those of English speakers with CAS, which led to further adaption of the DEMSS to the Swedish-speaking population [39]. Two survey studies were conducted to understand the clinical features, diagnostic criteria, assessment, and treatment of Iranian speakers with CAS [54, 55]. There was a consensus among Iranian SLPs for nine speech characteristics in Iranian speakers with CAS that are consistent with those found in English speakers with CAS. The authors concluded that these speech characteristics are clinically appropriate to inform CAS diagnosis in Iranian-speaking children [54]. Overall, Iranian SLPs used the methods commonly reported in the English literature for assessment, but the treatments they implemented lacked an evidence base [55]. CAS features in Cantonese, a tonal language without stress patterns, were explored using a modified Delphi survey [56]. Given that SLPs in Hong Kong are trained to apply the English clinical literature to assess, diagnose, and treat Cantonese-speaking patients with communication disorders [56], the applicability of the English clinical features to Cantonese was surveyed. An expert panel identified 79 clinical features that were reported in Cantonese speakers with CAS, with 29 clinical features considered to be important in making a differential diagnosis. In particular, lexical tone errors and difficulty in perceiving tones were identified as clinical features that had never been reported for English speakers with CAS. Further investigation of tone perception and production skills, as well as the development of a potential diagnostic tool, were then carried out, resulting in an increased understanding of CAS in Cantonese, and probably other tonal languages [32, 33].

### CAS in Cantonese-speaking populations

Cantonese is the main spoken language in Hong Kong. It is a syllable-timed tonal language with six contrastive lexical tones, comprising a high-level tone (tone 1), a high-rising tone (tone 2), a mid-level tone (tone 3), a low-falling tone (tone 4), a low-rising tone (tone 5), and a low-level-tone (tone 6) [57]. The tones impact lexical meanings. For example, /jɐu4 sœy2/ means “swim” and /jɐu4 sœy3/ means “convince.” These two verbs share identical segmental features and sequences; only the tones differentiate their meanings. Segmentally, Cantonese has 19 initial consonants, six final consonants, 11 vowels, and 11 diphthongs. Its within-syllable phonotactic structures include V, CV, and CVC. Words in Cantonese range from monosyllables to multisyllabic words.

Direct investigation of CAS in Cantonese speakers is scarce. In a recent scoping review, a total of four studies with a focus on Cantonese CAS were identified [58]. Wong [42] demonstrated the efficacy of a combined treatment approach of the syllable repetition method and modified DTTC therapy with two Cantonese speakers with CAS. A list of 29 clinical features that gained consensus among a group of experts for CAS diagnosis was proposed from a survey study [56]. When the lexical tone perception and production skills of Cantonese-speaking children with CAS were examined in another study [33], the results showed that lexical tone perception deficits were present in two out of three children with CAS, while all three children had lexical tone production difficulties. The authors concluded that lexical tone production was more universally affected than lexical tone perception in their sample of three children with CAS. Finally, the tone production and sequencing skills of these three children with CAS were investigated [32]. They found that the participants had difficulty varying fundamental frequency within syllables to form the high-rising tone (i.e., Cantonese tone 2), but had no problem with the low-falling tone contour (i.e., Cantonese tone 4) or the high-level tone (i.e., Cantonese tone 1), compared with age-and-gender matched children with typical development and children with both speech sound disorders (SSDs) and coexisting language disorders. In addition, the results showed that the CAS and control groups were significantly different in tone accuracy, tone consistency, and acoustic repetition duration in the context of the tone sequencing task (TST). The authors suggested that the TST might be a potential tool to differentiate children with and without CAS. Moreover, the effects of linguistic elements on pitch-variation skills were examined [32]. The results showed that the CAS and control groups were not different on tone accuracy, consistency, or repetition duration within groups when stimuli with different syllable structures and lexical status were involved. Due to the extremely small samples in these studies, further investigation was suggested to elucidate the relationship between these elements and motor speech control.

### Purpose of study

In addition to limited evidence about CAS in Cantonese speakers in terms of clinical features, potential diagnostic tools, and treatment strategies, the clinical practices of SLPs who work with Cantonese speakers were also unknown. It was unclear how these SLPs assess, diagnose, and treat children with CAS. The purpose of this study was to examine the clinical practice of SLPs in Hong Kong in assessing, diagnosing, and treating Cantonese speakers with CAS.

## Materials and methods

This study was approved by the PolyU Institutional Review Board (Reference number: HSEARS20210125012). The participants provided electronic consent before the start of the questionnaire. Participants agreed to participate by clicking the button indicating “Yes I agree to participate.” Subsequently, the survey questions were shown on the next page. Participants who clicked the button indicating “No, I do not wish to participate” were directed to the submission page.

### Survey design

This study was a voluntary open survey for qualified SLPs who were currently working with Cantonese speakers. The questionnaire was designed using a Google form by the first author, who is a doctoral student and an experienced, qualified SLP. The content of the questionnaire was reviewed by the second and third authors, who are SLPs and researchers working with children and/or adults with motor speech disorders. S1 Appendix presents the questionnaire. The questionnaire consisted of four sections, including demographic information, knowledge and experience about Cantonese speakers with CAS, approaches to assessment and diagnosis, and treatment strategies. There were eight, nine, thirteen, and eighteen questions in these sections, respectively, with a total of 48 questions. All of the questions were set as mandatory to allow for a completeness check. The questions were displayed on nine pages, including the introduction and informed consent on the first page and demographic information on the second page. The third page included a question about the number of children with CAS or suspected CAS that the participants had worked with. If the option “0” was selected, the system directed the respondent to the last page, which was the submission page. If other options were selected, the questionnaire was continued. On the fourth page, a question about the number of children who were initially diagnosed or suspected with CAS by the respondents was given. If the option “0” was selected, the system skipped the fifth page (assessment and diagnosis) and directed the respondent to the treatment section, which ran from the sixth page to the eighth page. The ninth page was the submission page. The questions in each section were presented in the same order for every participant. A back button was placed on each page to allow the respondents to change their answers at any time before submitting their responses. No summary of responses was given. There was no time limit for completing the questionnaire. The system saved the entries automatically and allowed the participants to continue at any time. The estimated time for completing the questionnaire was about 20 minutes.

### Procedure

Qualified SLPs who were currently working with Cantonese speakers were invited to participate in this study without any incentives offered. The questionnaire was distributed across the local SLP community by posting the link on the website and newsletters of the Hong Kong Association of Speech Therapists, the largest local professional organization of SLPs in Hong Kong. It was also posted on professional and personal social media accounts and Whatsapp chat groups. In addition, local universities forwarded the questionnaire link to alumni and in-person invitations were also issued. The questionnaire was open from January 2021 to August 2021, to reach as many local practicing SLPs as possible. Participants received the direct hyperlink to the Google form. Information about the research was shown on the first page and informed consent was obtained from the participants at the bottom of that page. The system recorded all submissions.

### Statistical analysis

All received and completed responses were analyzed. All data were entered into IBM^®^ SPSS^®^ Statistics for Macintosh, Version 28.0 [59] for data analyses. The respondents were divided into two groups, in some analyses, according to their years of clinical experience. Less experienced SLPs had less than ten years of clinical experience, while more experienced SLPs had ten or more years of clinical experience. This cut-off was based on the standard for CAS expertise among Chinese SLPs [58]. In order to examine the contributing factors to self-descriptions of CAS understanding levels, correlation analyses were performed. A chi-square test was used to explore the relationship between the experience levels of the SLPs (less versus more experienced) and their self-descriptions of their levels of understanding of CAS, between the experience levels and the types of training, between types of previous CAS training and clinicians’ self-descriptions of their levels of understanding of CAS, and between number of children with CAS they have worked with and their self-descriptions of their levels of understanding of CAS. Spearman’s rank correlation coefficient was used for identifying relationships between number of types of previous CAS training and clinicians’ self-described understanding and between years of clinical experience and self-descriptions of levels of understanding of CAS. Pearson correlation was used for identifying relationships between years of clinical experience and number of diagnostic features used, and between years of clinical experience and number of treatment approaches used. The level of significance adopted in this study was 0.05. Qualitative analyses were also conducted for questions that required free-text answers.

## Results

Eighty-three responses were received. After deleting duplicate entries, i.e., those with identical content that were received by the electronic system at the same time or within 20 minutes of each other, seventy-seven responses were involved in the analyses. No information was available about the view, participation, or completion rates.

### Demography of participants

All of the respondents were SLPs in Hong Kong. Table 1 presents the demographics of the respondents. The respondents’ years of clinical experience ranged from one month to 26 years, with a mean of 5.77 (SD = 6.16) years. There were 59 less experienced SLPs (mean years of clinical experience = 2.79; SD = 2.22) and 18 more experienced SLPs (mean years of clinical experience = 15.53; SD = 4.61) among the respondents.

**Table 1:** Demography of participants.

	Frequency (n = 77)	Percentage (%)
**Professional education**		
• Bachelor’s degree	46	59.7
• Master’s degree	31	40.3
**Place of professional training**		
• Hong Kong	76	98.7
• English-speaking country	1	1.3
**Language**		
• Cantonese	77	100
• English	77	100
• Mandarin	63	81.8
• Other languages (e.g., Korean, Spanish, or French)	2	2.6
**Language options offered to clients**		
• Cantonese only	45	58.4
• Cantonese and English	17	22.1
• Cantonese, English, and Mandarin	15	19.5
• Cantonese and other languages	0	0
**Post-qualification education**		
• None	46	59.7
• Master’s degree	30	39
• Doctoral degree	1	1.3

**Fig 1:**
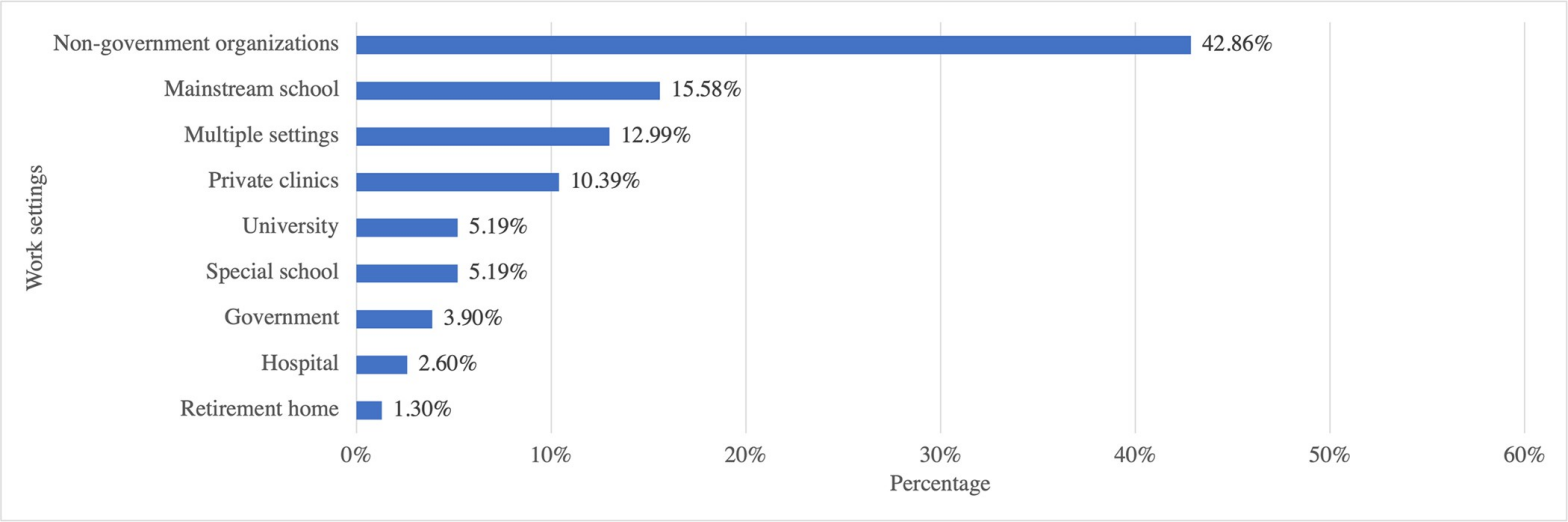
Distribution of respondents’ work settings.

Fig 1 presents the distribution of work settings among the respondents. Thirteen percent of respondents worked in multiple settings, such as a private clinic and a hospital; a mainstream school and a university; a mainstream school, a private clinic, and a retirement home; etc.

### Knowledge and experience of SLPs working with Cantonese speakers with CAS

**Fig 2:**
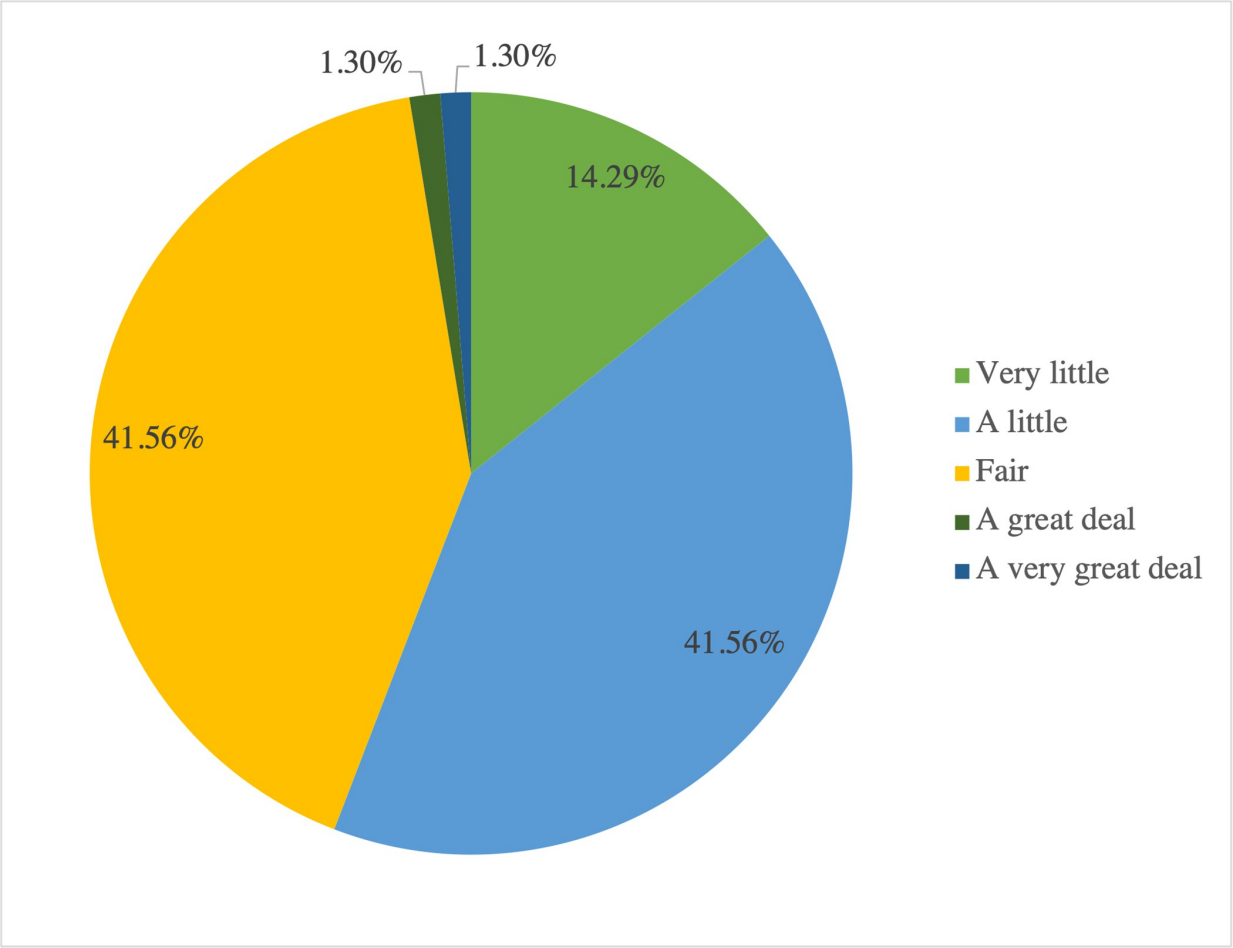
Respondents’ self-descriptions of their level of understanding of childhood apraxia of speech.

Nine questions relating to the knowledge and experience of SLPs working with Cantonese speakers with CAS were included. Fig 2 presents the results of respondents’ self-descriptions of their levels of understanding of CAS, which were primarily “a little” or “fair.” Though there was no significant association between experience levels (less or more experienced) and self-described understanding of CAS, *X*^*2*^ (4, 77) = 7.135, *p* = 0.13, there was a significant but weak association between the years of experience and self-described understanding of CAS in Cantonese speakers, *r*_*s*_ = 0.272, *p* = .02. SLPs with more years of experience indicated they had a better understanding of CAS in Cantonese speakers when compared with self-descriptions by SLPs with less years of experience.

**Fig 3:**
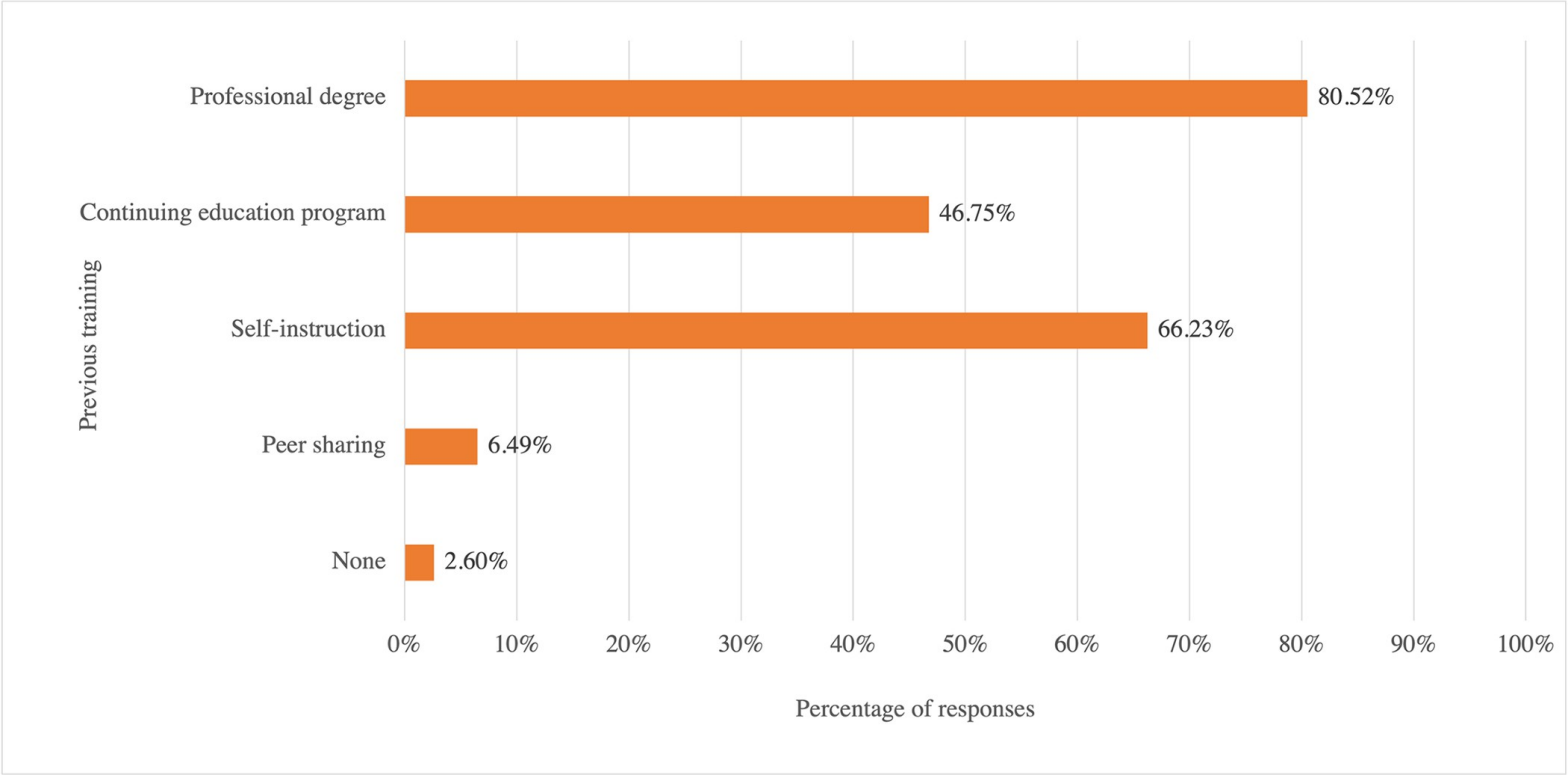
Respondents’ previous training in childhood apraxia of speech.

Fig 3 presents the respondents’ previous training in CAS. Many had learned about CAS through more than one source; two reported no training at all. There was a significant association between the types of previous training on CAS and the self-described level of understanding of CAS in Cantonese speakers, *X*^*2*^ (40, 77) = 108.974, *p* < 0.001. There was a weak positive association between the number of types of previous training and the self-described level of understanding of CAS in Cantonese speakers, *r*_*s*_ = 0.396, *p* < .001. Any combinations of professional degree, continuing education program, and self-instruction were associated with a higher level of understanding of CAS.

Four follow-up questions were asked to further examine how much the aforementioned four types of training contributed to the respondents’ understanding of CAS. Among the 77 responses, 31 responses were excluded due to incomplete or inappropriate entries (e.g., the sum of the four percentages was not 100), resulting in 46 usable responses. The descending order of the amount of CAS training from each type of source among the respondents was professional degree (mean = 47.3%; SD = 30.8%), self-instruction (mean = 26.2%; SD = 21.9%), continuing education program (mean = 21.6%; SD = 25.2%), and peer sharing (mean = 4.9%; SD = 11.0%). The results of further analysis of the relationship between years of experience and prior CAS training showed that more experienced SLPs (n = 11) obtained the most CAS training from continuing education programs (mean = 45.0%; SD = 14.1%), followed by their professional degree programs (mean = 24.5%; SD = 18.1%), self-instruction (mean = 20.9%; SD = 17.1%), and others, such as peer sharing (mean = 9.54%; SD = 12.1%). Less experienced SLPs (n = 35) obtained the most CAS training from their professional degree programs (mean = 54.4%; SD = 30.5%), followed by self-instruction (mean = 27.9%; SD = 23.0%), continuing education programs (mean = 14.3%; SD = 23.3%), and others, such as peer sharing (mean = 3.4%; 10.1%). There was no significant relation between the level of clinical experience (i.e., less or more experienced SLPs) and the types of training in CAS, *X*^*2*^ (4, N = 77) = 10.202, *p* = 0.42.

**Fig 4:**
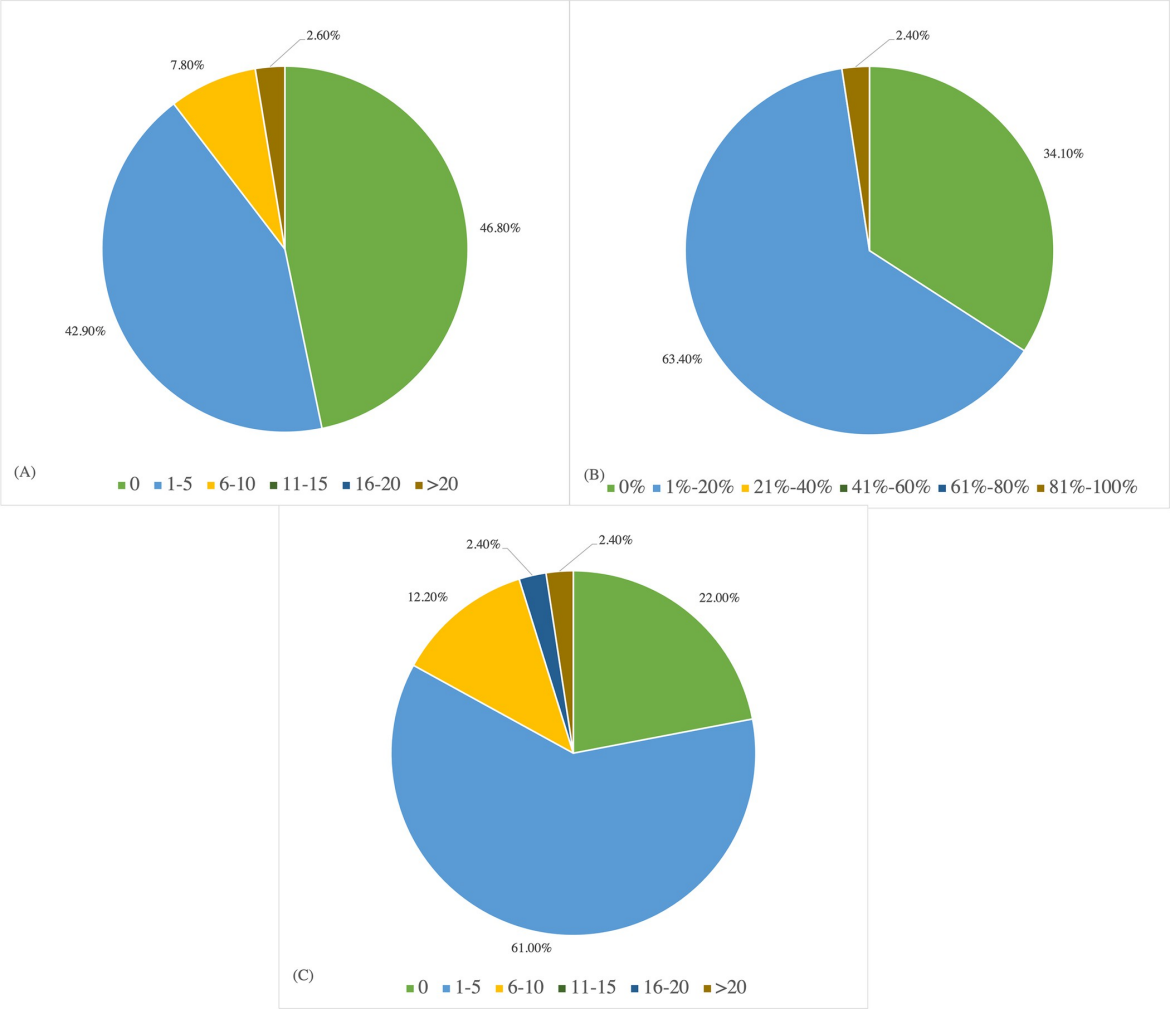
Distribution of Cantonese-speaking children with or suspected with childhood apraxia of speech on the respondents’ caseloads. (A) Distribution of the number of Cantonese-speaking children with or suspected with childhood apraxia of speech on the respondents’ caseloads; (B) Distribution of percentage of Cantonese speakers with or suspected with childhood apraxia of speech on the respondents’ caseloads; (C) Distribution of the number of children with or suspected with childhood apraxia of speech who were initially diagnosed or suspected by the respondents.

Fig 4 presents the caseloads reported by the 77 respondents. Fig 4A presents the distribution of the number of Cantonese-speaking children with CAS or suspected CAS (sCAS) in the respondents’ caseloads. Slightly more than half of the respondents reported having experience with Cantonese speakers with CAS and sCAS but few had worked with more than five such clients. There was no significant relation between experience with CAS (having seen any children with CAS or not) and their self-described level of understanding of CAS, *X*^2^ (4, *N* = 77) = 5.973, *p* = 0.20, but a significant association was found between the number of children with CAS they had worked with and their self-described level of understanding of CAS, *X*^2^ (12, *N* = 77) = 47.251, *p* < 0.001; more experience resulted in better understanding.

Fig 4B illustrates the distribution of percentages of Cantonese speakers with CAS or sCAS on the 41 respondents’ current caseloads. Among the 27 respondents who were working with Cantonese speakers with CAS or sCAS, work settings included NGOs (10/27), private clinics (6/27), multiple settings (4/27), universities (3/27), special schools (2/27), mainstream schools (1/27), and government departments (1/27). There was no respondent who was working at a hospital or a retirement home who reported CAS cases on their caseload.

Fig 4C presents the distribution of the number of children with CAS or sCAS who were initially diagnosed or suspected of having CAS by the 41 respondents. The largest proportion had diagnosed or suspected CAS in one to five children.

### Assessment

**Fig 5:**
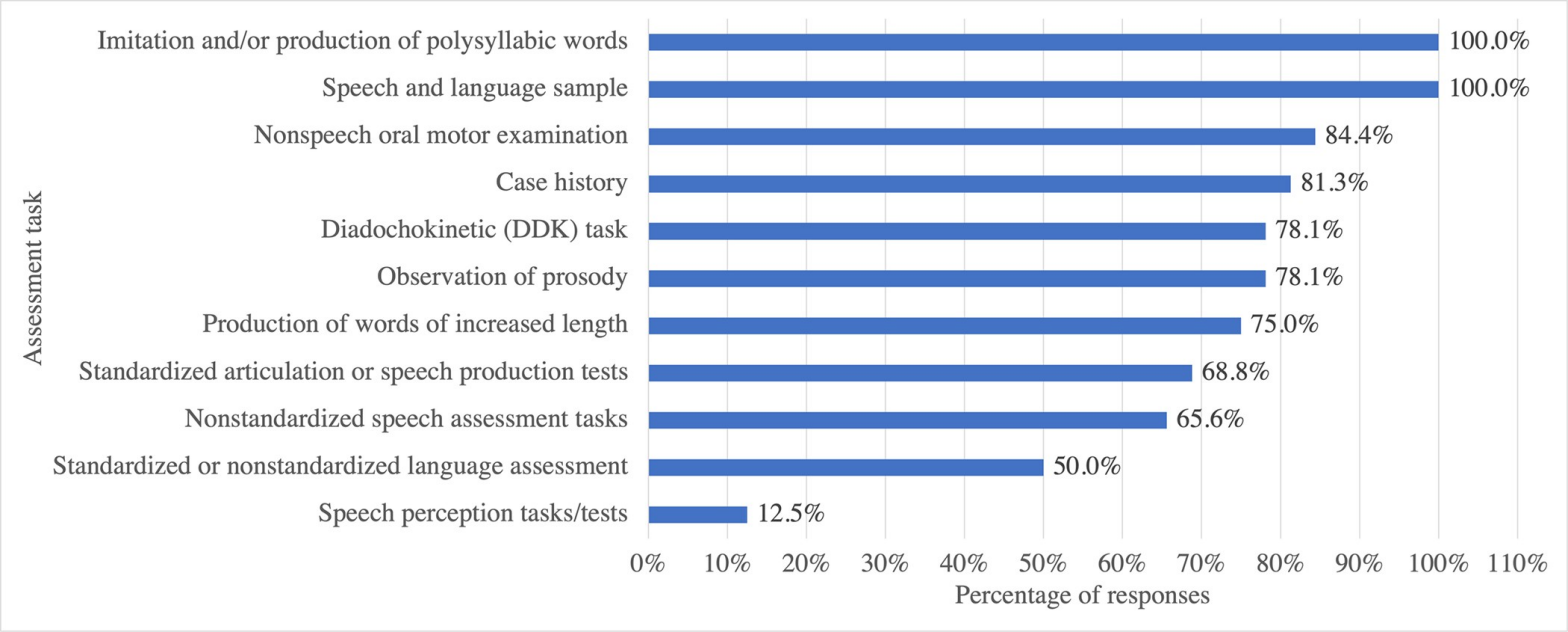
Percentage of respondents using specific assessment tasks for Cantonese speakers with or suspected with childhood apraxia of speech.

The thirty-two respondents (n = 32) who had diagnosed or suspected CAS in Cantonese speakers completed the following two sections on assessment and diagnostic features and criteria, which included three questions related to assessment methods, three questions related to diagnostic processes, and seven questions related to CAS clinical features. Fig 5 illustrates the percentage of the respondents using each diagnostic task to assess Cantonese speakers with CAS or sCAS. All respondents collected speech and language samples and asked the children to imitate or produce polysyllabic words. Nonspeech oral motor examinations, case histories, diadochokinetic (DDK) tasks, observations of prosody, and increasing word length tasks were also commonly used. The use of speech perception tasks or tests was rare.

Two follow-up questions regarding information about any standardized assessment tools and objective/quantitative measures or tasks that were used by the respondents were asked. None of the respondents reported the use of standardized tests or quantitative measures that are specified for CAS diagnosis.

### Diagnostic clinical features and criteria

The participants were asked to report their use of any clinical checklists in assisting with the diagnostic process for Cantonese speakers with CAS. There were twenty respondents (62.5%; 20/32) who used only their own clinical checklists to aid in the CAS diagnostic process. Five respondents (15.6%; 5/32) used existing checklists only: three respondents used Strand’s 10-point checklist [10], one respondent used ASHA’s three consensus features [2], and one respondent used Wong’s 29 clinical features [56]. Four respondents (12.5%, 4/32) reported that they did not use any clinical checklists for making a CAS diagnosis. Three participants (9.4%, 3/32) responded that they used both existing and their own clinical checklists in their clinical practices. The remaining three responses did not specify what they used.

The respondents were asked to list all the clinical features they used to diagnose CAS in Cantonese-speaking children. There were 20 different sets of clinical features submitted by the 23 respondents; three did not provide their checklists. The participants either used their own clinical checklists only (20 respondents) or used both their own and existing checklists (3 respondents) to aid in the CAS diagnostic process. No identical sets of clinical features were reported. Table 2 summarizes the clinical features and the diagnostic criteria that were used by those 20 respondents. There were 27 different clinical features used. Three clinical features were used by more than 50% of the respondents, namely “nonspeech or speech groping” (85%; 17/20), “inconsistent errors” (75%; 15/20), and “increased errors on increased syllable or sentence length” (55%; 11/20). Four clinical features were used by more than 25% or more of the respondents, including “poor DDK performance” (40%; 8/20), “more difficulty in volitional speech or non-speech movements than in automatic counterparts” (35%; 7/20), “reduced accuracy on sound or word imitation” (30%; 6/20) and “articulatory errors” (25%; 5/20). There was no significant correlation between the SLPs’ years of clinical experience and the number of features they used, *r*_*s*_ = 0.080, *p* = .74.

**Table 2:** Summary of the clinical features used by 20 speech-language therapists in Hong Kong for childhood apraxia of speech diagnoses in Cantonese speakers.

		1	2	3	4	5	6	7	8	9	10	11	12	13	14	15	16	17	18	19	20
Clinical features																					
1	Nonspeech or speech groping	✓		✓	✓			✓	✓	✓	✓	✓	✓	✓	✓	✓	✓	✓	✓	✓	✓
2	Inconsistent errors	✓	✓	✓	✓				✓	✓	✓	✓	✓	✓	✓	✓	✓		✓	✓	
3	Increased errors on increased syllable or sentence length	✓		✓		✓	✓		✓					✓	✓	✓		✓		✓	✓
4	Poor DDK performance	✓		✓		✓	✓								✓	✓			✓		✓
5	More difficulty on volitional speech/non-speech movements than on automatic counterparts			✓		✓			✓		✓			✓					✓		✓
6	Reduced accuracy on sound/word imitation		✓	✓	✓			✓									✓			✓	
7	Articulatory errors (e.g. sound distortion, anticipatory errors, perseverative errors, or uncommon speech sound errors)		✓	✓			✓		✓								✓				
8	Discrepancy between verbal receptive and expressive language skills	✓		✓												✓				✓	
9	Poor nonspeech oral motor skills		✓			✓	✓												✓		
10	Tone production errors	✓		✓	✓						✓										
11	Limited phonetic inventory	✓														✓		✓			
12	Abnormal prosody	✓		✓								✓									
13	Deficits in coarticulation (transition between phonemes or syllables, for example, syllable segregation)											✓					✓	✓			
14	More struggle with speech movements than non-speech movements												✓			✓					
15	Reduced consistency on increased syllables or sentence length				✓						✓										
16	Difficulty in imitation of polysyllabic words											✓							✓		
17	Vowel or diphthongs errors			✓												✓					
18	Reduced speech rate		✓																		
19	Poor gross and fine motor skills		✓																		
20	Errors on aspirated sounds			✓																	
21	Difficulty in initiating speech			✓																	
22	Intrusive schwa			✓																	
23	Aware of errors but unable to correct them			✓																	
24	Slow progress in articulation treatment							✓													
25	Low speech intelligibility									✓											
26	Better performance on repetitions													✓							
27	Limited speech																	✓			
Criteria for CAS diagnoses		N	6/6	8/15*	3/5	3/4	3/4	3/3	4/5^#^	3/3	3/5	5/5	3/3	3/5	N^^^	4/8*	5/5*	N	5/6	N	N

*Note*. The criteria for CAS diagnoses differed across the respondents. N indicates that the respondent did not specify that criterion

* indicates that the clinical features should be observed across 2–3 different tasks

^#^ indicates that 3 out of 4 speech features and 1 out of 2 non-speech features should be observed

^^^ indicates that the respondent used a 5-point Likert scale to perceptually rate the clinical features, which ranged from “all the time”, “usually”, “sometimes”, “occasionally” to “none”

Abbreviations: CAS = childhood apraxia of speech; DDK = diadochokinesis; N = not clear or not specified

The diagnostic criteria reported by the respondents varied. The respondents who used the existing clinical checklists adopted the suggested criteria, such as “4 out of 10 clinical features across three or more different tasks” for Strand’s 10-point checklist [10]. For those who used their own checklists, the diagnostic criteria were categorized as requiring the presence of all features (30%; 6/20) or requiring more than 50% of features (45%; 9/20). In addition, there were four criteria that required observation of features across two to three different tasks (20%; 4/20) while the others did not specify. One respondent reported the use of a 5-point Likert scale on the four clinical features, without reporting the exact criteria.

### Importance of clinical features

**Fig 6:**
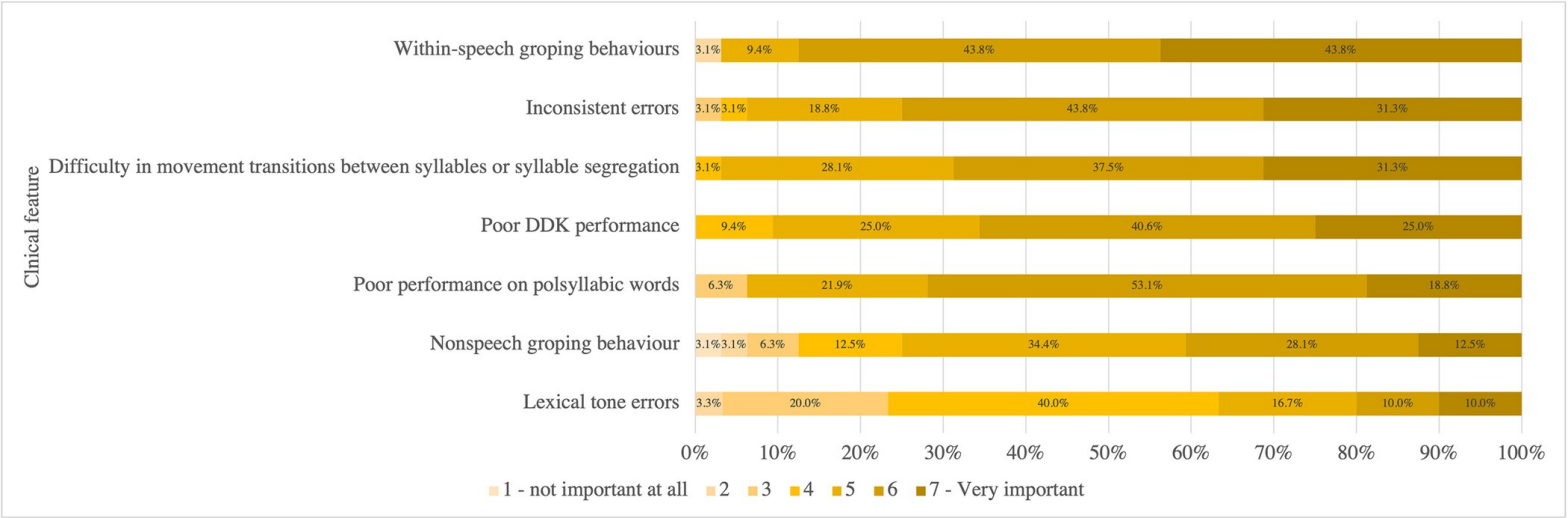
Distribution of ratings of seven clinical features for making a differential diagnosis of childhood apraxia of speech.

A 7-point Likert scale, ranging from “1-not important at all” to “7-very important” was used for rating the importance of seven clinical features that have been reported for making a differential diagnosis of CAS in Cantonese speakers. Fig 6 illustrates the distributions of the ratings. The means and standard deviations are presented in Table 3.

**Table 3:** Descriptive statistics of ratings of seven clinical features of childhood apraxia of speech for making a differential diagnosis.

Clinical feature	Mean	Standard deviation
Poor performance on polysyllabic words	6.78	0.97
Within-speech groping behaviors	6.12	1.01
Inconsistent errors	5.97	0.67
Difficulty in movement transitions between syllables or syllable segregation	5.97	0.86
Poor diadochokinesis (DDK) performance	5.81	0.93
Nonspeech groping behaviors	5.06	1.41
Lexical tone errors	4.25	1.39

### Treatment

Forty-one respondents (n = 41) completed the section on treatment. Questions included structure of delivery (frequency of treatment, duration of each treatment session, and time allocated for speech production practice), treatment focus, and treatment approach.

#### Structure of delivery

**Fig 7:**
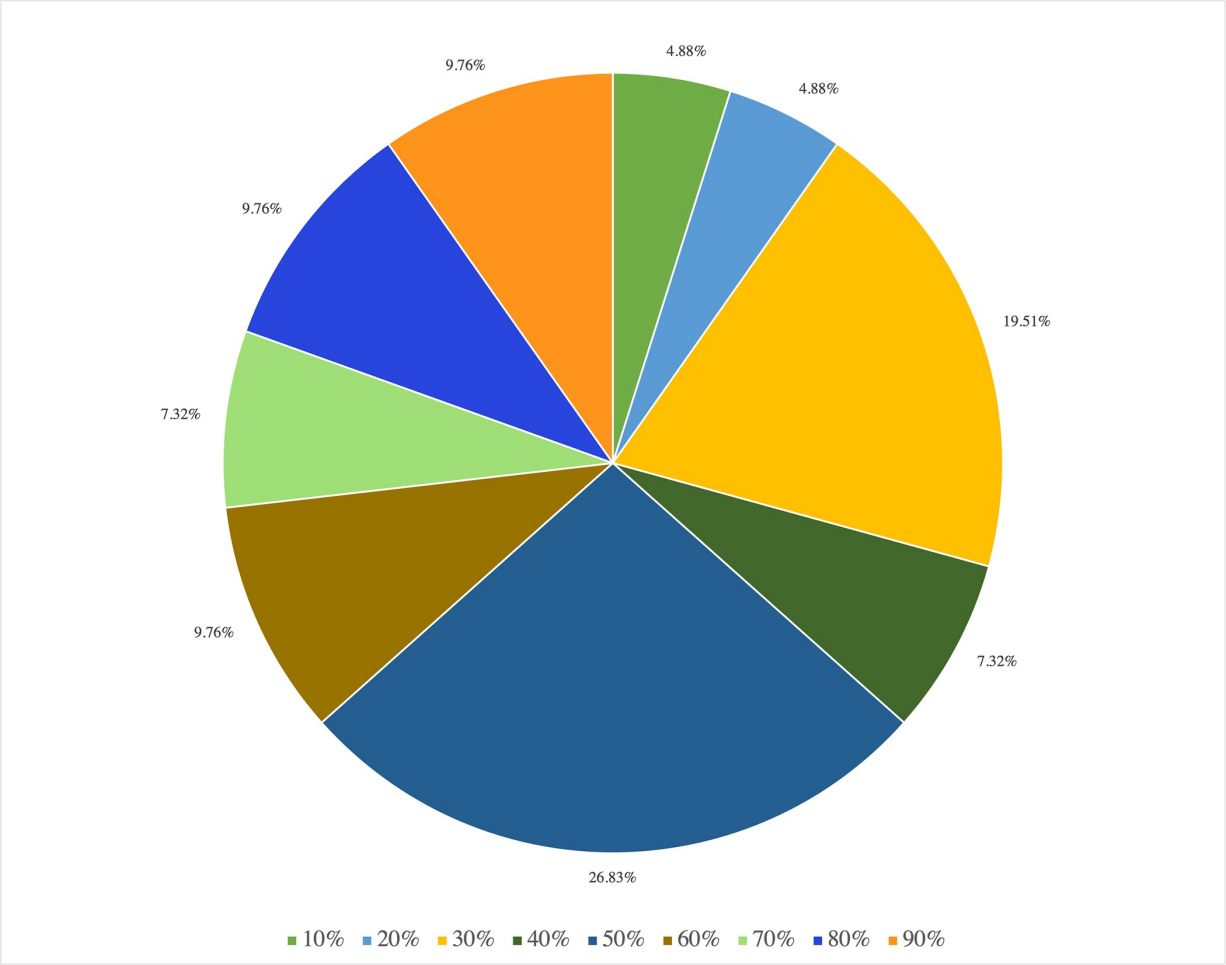
Percentage of respondents’ time allocated to speech production practice in treatment sessions.

Most of the respondents provided treatment to children with CAS less than once a week (43.9%; 18/41), followed by once a week (41.5%; 17/41) and twice a week (14.6%; 6/41). No respondents provided treatment more than twice a week. In terms of treatment duration, most of the treatment sessions were 35 to 50 minutes (46.3%; 19/41), followed by 30 to 34 minutes (22%; 9/41), less than 30 minutes (17.1%; 7/41), and 51 to 60 minutes (12.2%; 5/41). Fig 7 presents the estimated percentage of speech production practice in the sessions. The most common practice was to spend about half of each session working on speech.

Only 14.6% (6/41) of the respondents provided between-session block treatment (i.e., session[s] focused on speech production followed by session[s] dedicated to other goals). Five respondents reported one, three, four, eight, and 18 sessions for the speech production practice block. One respondent responded with “zero session”, which was considered to be an error. With respect to other targets, two respondents scheduled two sessions, one respondent scheduled four sessions, and another respondent scheduled eight sessions per block. Two responses were considered to be errors as “zero session” was entered. Regardless of the session allocation, almost every respondent targeted language skill as the one other goal (92.7%; 38/41). The other treatment areas included speech perception skills (26.8%; 11/41), nonspeech oral motor abilities (26.8%; 11/41), social skills (17.1%; 7/41), and literacy skills (4.9%; 2/41).

#### Treatment focus

Regarding the selection of targets, most of the respondents (78%; 32/41) first targeted segments (e.g., phoneme and syllable structure accuracy) in their treatment of speech production abilities, followed by both segments and suprasegmentals (17.1%; 7/41). Only two respondents (4.9%; 2/41) targeted suprasegmentals alone from the beginning of treatment.

**Fig 8:**
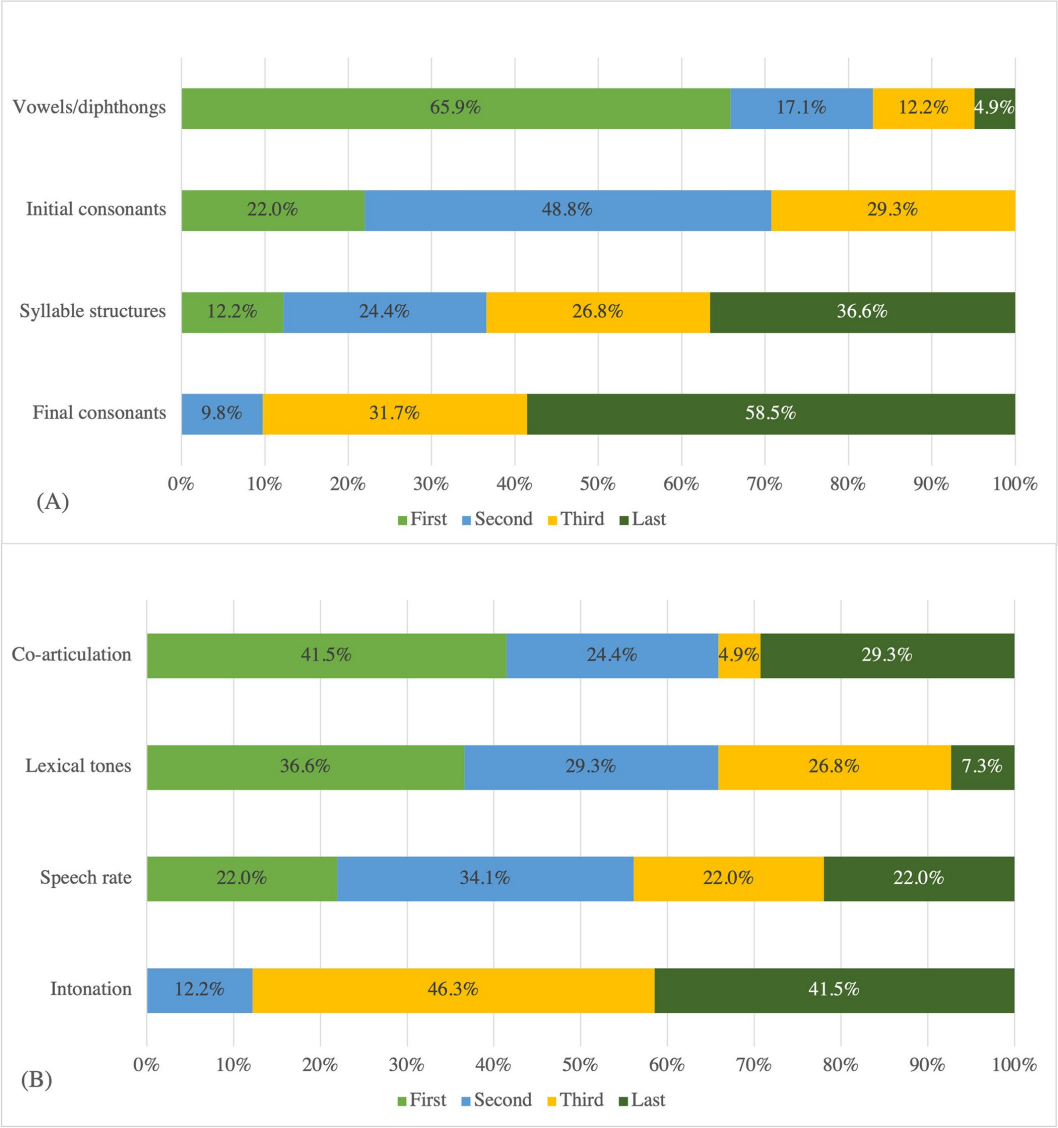
Distribution of treatment priorities for different types of segments and suprasegments.

Fig 8 presents the treatment priorities for different types of segments and suprasegmentals reported by the 41 respondents. With respect to segments, most respondents targeted vowels or diphthongs, followed by initial consonants. The respondents’ reasons, for this included: 1) remediation of vowels/diphthongs and initial consonants impacts clients’ intelligibility, 2) vowels/diphthongs and initial consonants are typically acquired earlier, according to developmental data, and 3) the targets were selected based on stimulability. With respect to suprasegmentals, most respondents target coarticulation skills, followed by lexical tones. Reasons for this included 1) coarticulation and lexical tones have more impact on clients’ intelligibility and 2) lexical tone errors affect the meanings of words.

#### Treatment approaches

**Fig 9:**
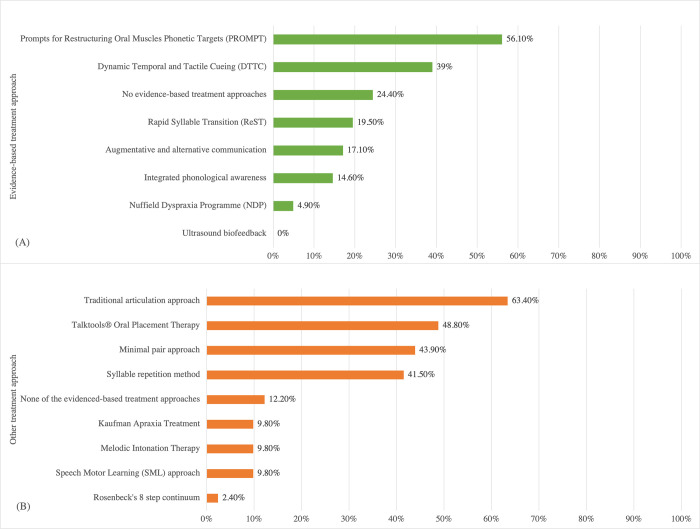
Respondents’ use of evidence-based and other treatment approaches for children with childhood apraxia of speech. (A) Distribution of evidenced-based treatment used by the respondents. (B) Distribution of other treatment approaches used by the respondents.

Fig 9 summarizes the respondents’ use of evidence-based and other treatment approaches for children with CAS. Fig 9A illustrates the distributions of evidenced-based treatments used. Ten respondents did not use any evidence-based treatment approaches. PROMPT was the most popular, followed by DTTC and ReST. There was no significant relation between the years of clinical experience and the number of evidence-based treatment approaches used, *r* = 0.013, *p* = 0.93.

Other approaches that have no or limited evidence for use with children with CAS are presented in Fig 9B. Many clinicians were still using several of these approaches. There was no significant relation between the years of clinical experience and the number of non-evidence-based approaches being used, *r* = 0.234, *p* = 0.14. There also was no significant relation between the years of clinical experience and the number of treatment approaches used, irrespective of evidence level, *r* = 0.186, *p* = 0.24.

Thirty-one responses were received on a follow-up question that required rating the level of implementation of the evidence-based treatment approaches on a 7-point Likert scale (i.e., 1 for minimal implementation and 7 for full implementation). The mean rating was 4.26 (SD = 1.69). There was no significant relation between the years of clinical experience and the degree of implementation of evidence-based treatment approaches, *r* = -0.212, *p* = 0.25.

## Discussion

The purpose of this study was to examine the current clinical practices of SLPs in assessing, diagnosing, and treating Cantonese speakers with CAS in Hong Kong.

### Principal results

#### Knowledge and experiences of CAS

The results indicate that the understanding of CAS among Hong Kong practicing SLPs still requires attention. Generally, SLPs in Hong Kong report having “a little” or “fair” understanding of CAS. Given that there have been few investigations of Cantonese speakers with CAS [58] and insufficient numbers of trainings or other resources for self-instruction to equip the clinicians, the local clinicians can only rely on their actual clinical experience. As CAS is not a commonly seen speech sound disorder and about half of the respondents (46.8%) had never worked with any children with CAS, the general understanding among clinicians is limited. The clinicians’ self-reported understanding levels increased gradually when they worked with more children with CAS or received more types of CAS training. These results differ from those of the survey study that found that about 60% of SLP respondents in the US had moderate or high levels of confidence in their ability to make a differential diagnosis of CAS [11]. More research and transfer of knowledge to clinical practice are needed.

While the level of understanding of CAS is affected by clinical experience, work settings play an important role. SLPs who work in NGOs or private clinics have large and varied client bases and may see children with CAS in some preschool services. For example, the respondent who had seen more than 20 children with CAS was working in a private clinic. Moreover, it is unsurprising that SLPs in settings with more typical client bases, such as mainstream and special schools, have relatively less exposure to children with CAS. Exposure to children with CAS in governmental settings varied, ranging from one to five to more than 20 children with CAS. SLPs in the Department of Health in Hong Kong mainly provide public assessment services to children who are under twelve years of age [60]. The SLPs in this setting may see children with CAS in their daily clinical work due to wide exposure to different communication disorders in children. It is important to note that the numbers of CAS cases that the SLP respondents had seen were self-reported; thus, these data depend on the respondents’ understanding of CAS.

#### Assessment

Results showed that the respondents did not use any standardized assessments that were specifically designed for children with CAS. This is unsurprising because there are no valid and reliable assessment tools available for Chinese speakers with CAS [58].

There was a strong tendency for using seven assessment tasks among the SLPs in Hong Kong, namely imitation and/or production of polysyllabic words, speech and language samples, nonspeech oral motor examinations, case history collection, DDK tasks, observation of prosody, and production of words of increasing length. The use of these tasks is aligned with the standards suggested for assessment of older and less severe English speakers with CAS [61]. However, the actual manner of clinical implementation of these tasks remains unknown. This is because the clinicians were not asked for detailed descriptions of the identified tasks with respect to the content, administrative procedures, and interpretation processes that they used. It is unknown whether the clinicians implement the English tasks with Cantonese-speaking children with linguistic modifications, given that exact adaptation may be inappropriate due to the different clinical manifestations of CAS in different languages [56].

The use of objective/quantitative measures by the local clinicians in their CAS assessments is uncommon. In this sense, there is a gap between research and clinical practice. There are three possible reasons for this. First, although objective/quantitative measures such as MPT [8] and SRT [9] have been reported as clinically useful measures for English speakers with CAS [62], criterion-referenced standards for these measures have not yet been reported for Cantonese speakers with CAS. Without any valid data from Cantonese speakers with CAS, it is unsurprising that these tasks are not used locally. In addition, SLPs in Hong Kong prefer tasks that provide information on both speech and language abilities, as compared with tasks that provide information about a single aspect such as speech production only. This is illustrated by the 100% usage of speech and language samples among the respondents. This may also be due to time constraints on assessment sessions. Generally, only one assessment session that lasts for about an hour will be conducted in most local clinical settings. A speech and language sample that provides information about the client’s optimal level of communication is cost-effective in this situation. However, local SLPs do understand the importance of examining underlying deficits. Therefore, tasks that are short but informative about speech motor control, such as DDK tasks, were also selected by most of the respondents. In comparison, existing measures that have standardized procedures are not commonly used.

Moreover, some of the SLPs in Hong Kong have designed and used their own measures to collect information for CAS assessment. These measures include error consistency, accuracy of syllable shapes, and phrases of increasing length. These measures may have been designed based on clinical features reported in English speakers with CAS, such as increased inconsistency and difficulty with longer or more complex syllables and word shapes (ASHA, 2007). However, the validity of these self-designed tasks is unknown, and importantly, the interpretation of the results may vary across clinicians. Therefore, there is an urgent need to investigate the validity of these tasks as well as to investigate the use of objective/quantitative measures with Cantonese-speaking children with and without CAS to inform clinical practice.

#### Diagnostic clinical features and criteria

Perceptual judgment of clinical features is the most popular approach for diagnosing CAS among Hong Kong SLPs, which is consistent with the current clinical practice in some other countries such as Iran [54]. A tendency of using “nonspeech or speech groping”, “inconsistent errors”, and “increased errors on increased syllable or sentence length” was reached for CAS diagnosis in Cantonese speakers. However, only the second and third of these popular features appear to be valid in making a CAS diagnosis [4, 63] and their validity has not been proven for Cantonese speakers with CAS. This result further supports the claim that the understanding of CAS among local SLPs requires attention. The various uses of clinical features among local clinicians are consistent with the situation found for Cantonese-speaking adults with apraxia of speech [64], with respect to which the variety of clinical features used is problematic as this may raise concerns about the validity and reliability of apraxia diagnoses.

No one of the clinical checklists was commonly used in Hong Kong, even though a list of 29 clinical features, which have gained consensus among a group of local experienced SLPs, is available [56]. This is probably due to the absence of validity data and the potential ambiguity of some of its clinical features. For example, it is unclear how to determine “poor treatment progress” and “slow progress in learning new consonants and vowels”. It is also unclear how to compare cognitive skills and expressive language skills in order to determine whether the client has “better cognitive skills than expressive language skills.” Nevertheless, the list provides the basis for further development of a checklist for local clinical use. Apart from developing a clinical checklist for Cantonese speakers, investigation of the sensitivity and specificity of the existing checklists (e.g., Strand’s 10-point checklist) for Cantonese speakers may also inform clinical practice. Investigation of the individual clinical features listed in the checklists may also shed light on their validity. Given that the combination of syllable segregation, lexical stress matches, accuracy of polysyllabic word production, and DDK performance has high diagnostic accuracy for English-speaking children with CAS [4], investigation of these clinical features for Cantonese speakers with CAS is also recommended.

Most of the respondents reported that CAS diagnoses could only be made upon detection of at least 50% of the clinical features listed in their own checklists. This reflects the belief that CAS diagnosis should not be made based upon any single clinical feature, but a combination of these. In addition, the respondents differed with respect to whether the clinical features should be observed once or across tasks in the assessment. Although no study has examined this difference, previous research has tended to require observation of multiple clinical features across multiple tasks in the context of English [10, 65] and Cantonese [32, 33]. Further investigation is recommended to determine whether it is necessary to observe clinical features across different speaking contexts.

#### Importance of clinical features

The results of respondents’ ratings on the importance of seven clinical features suggest that local SLPs are familiar with the literature and aware of the importance of the reported clinical features. Lexical tone errors were rated as neither important nor unimportant, which is consistent with the previous ratings of a group of local experts [56]. However, both acoustic and perceptual evaluations have recently shown that Cantonese speakers with CAS have difficulty in tone production that is not shared by children with non-CAS, SSDs and coexisting language disorders, or children with typical development [32, 33].

#### Treatment

There is a discrepancy between local and international clinical practice in terms of the structure of treatment delivery and the intervention approaches used. The session frequency reported by the local SLPs was much lower than the median of three times a week (maximum of once a day and minimum of twice a week) typically suggested for motor-based CAS treatment, while the typical Hong Kong session duration is consistent with the literature [3]. The frequency and duration of sessions are similar to those for services provided to children with SSDs, which are two to four times per month, with session durations ranging from 30 to 60 minutes [66]. Thus, local clinicians appear to treat CAS in the same way as other types of SSDs, although high treatment intensity has been recommended for children with CAS [3]. A variety of ways to vary treatment intensity have been suggested in the literature. For example, ReST therapy is recommended to be implemented in intensive therapy blocks (four sessions per week for three weeks) followed by a break from speech work for at least 6 weeks [67], but such strategies have not been adopted locally. In addition, treatment delivery schedules might be affected by SLPs’ confidence in their diagnoses. Since CAS is not a common diagnosis and many SLPs have never diagnosed or suspected CAS in any Cantonese speakers, modification of the once-a-week treatment model may not be seen as warranted for an uncertain diagnosis in many clinical settings.

Most of the SLPs in Hong Kong prioritize segmental accuracy over prosody in the treatment of children with CAS. This may be due to the imbalanced coverage of speech sound accuracy versus prosodic treatment methods in the literature. Treatments that had received the most attention up until the last few decades, such as the traditional articulation approach and minimal pairs intervention, focus more on segmental accuracy. This may drive clinicians to focus on segments first. The SLPs in Hong Kong work on vowels or diphthongs and initial consonants earlier than syllable structures and final consonants. The reasons provided by the respondents reflected that developmental sequences and intelligibility guide their treatment planning. Hong Kong SLPs prioritize coarticulation and lexical tones more highly than speech rate and intonation. The importance of lexical tone production in Cantonese was emphasized in their comments. This likely reflects the SLPs’ careful consideration of the linguistic differences between Cantonese and English, given that tone errors alter meaning while inappropriate speech rate and intonation do not [57, 68].

In addition to speech production skills, SLPs in Hong Kong also work on other aspects of communication such as language skills for children with CAS in the same or separate sessions. This reflects that these SLPs are aware of possible co-existing conditions in speakers with CAS and treat clients using a more whole-person approach. However, evidence-based treatment approaches that incorporate language issues (e.g., NDP and DTTC) or that allow blocks for language treatment (e.g., ReST) were not commonly used in Hong Kong. This may be due to the lack of modifications of these approaches for Cantonese speakers with CAS. Instead, the traditional articulation approach [69], Talktools® Oral Placement Therapy [27], and PROMPT [15] were used by more than half of the respondents. These results differ from the predominant clinical practices for children with CAS in English-speaking countries [50, 51] and other countries such as Iran, where oral motor exercises were mostly used [55]. The high use of the traditional articulation approach may be due to the plentiful availability of relevant clinical resources. The two approaches that require attendance at continuing education courses have a strong influence on clinicians’ practice [52]. Evidence for or against using traditional articulation approaches or Talktools® Oral Placement Therapy with speakers with CAS is needed.

Although some of the SLPs in Hong Kong do provide evidence-based treatments for children with CAS, the implementation of these programs is not thorough. The partial implementation of these treatments can be attributed to multiple possible factors such as limitations in session frequency, the presence of possible co-existing conditions in the clients, and less than a thorough understanding of the treatment approaches, all of which were reported by the respondents. As a result, the positive treatment outcomes demonstrated in treatment studies are not guaranteed. Nevertheless, the partial implementation of evidence-based treatment for children with CAS has also been found in English-speaking countries [51], suggesting a universal trend in the field of speech-language pathology and an urgent need for investigations of the effectiveness of these approaches in real clinical settings.

### Limitations and future investigations

As the first-ever study examining the clinical practice of CAS in Hong Kong Cantonese speakers, this study was limited in several ways. First, there were only 77 responses to this study. This is estimated to represent approximately 7% of the SLPs in Hong Kong [70]. The small sample size may reduce the power of the findings. In addition, with respect to the content of the questionnaire, the provision of specific choices may have resulted in bias.

The results of this study, especially the limited understanding that SLPs in Hong Kong have of Cantonese speakers with CAS, suggest that further investigations are urgently needed. Appropriate future directions include identifying clinical features of CAS in Cantonese speakers, assessment methods, and diagnostic standards, as well as treatment strategies. Studies focusing on Cantonese lexical tones in all of these respects are especially needed. Second, this study showed that continuing education programs played a role in the selection of treatment approaches for children with CAS among Hong Kong SLPs. Given that the content of most continuing education programs is not peer-reviewed [71], it is unknown how much evidence-based information is delivered through these courses. Further investigation of these continuing education programs may benefit the field. Last, the partial implementation of evidence-based treatment programs reveals the need for investigation of treatment effectiveness in real clinical settings.

## Conclusion

This survey study aimed to examine the clinical practice of CAS in Hong Kong. The level of understanding of CAS among local SLPs requires attention. There is no standardized assessment tool used clinically, but there is a strong tendency for using seven assessment tasks, including imitation and/or production of polysyllabic words, speech and language samples, and others. Perceptual judgment of clinical features is still the most popular approach. Although three features were commonly used (“nonspeech or speech groping”, “inconsistent errors”, and “increased errors on increase syllable or sentence length”), their validity is still unknown. Also, local SLPs are using treatment approaches that have limited evidence, in the context of less treatment frequency, targeting both speech and language skills within the same session, and partial implementation of the approaches. The results of this study suggest a strong need for future investigations of CAS in Cantonese speakers with respect to clinical features (especially lexical tone), assessment methods, diagnostic standards, and treatment efficacy and effectiveness.

## Supporting information

S1 Appendix Survey questions. (DOCX)
