# Supplementary material for: Clinical practice of childhood apraxia of speech in Hong Kong: A web-based survey study
Source: PLoS One. 2023 Apr 7;18(4):e0284109. doi: 10.1371/journal.pone.0284109 (PMC10081777; doi:10.1371/journal.pone.0284109)
Supplement: S1 Appendix — (DOCX) [file pone.0284109.s001.docx]

| S1 Appendix.  *Survey questions.* | |
| --- | --- |
| Background information, clinical experience, and knowledge | |
| 1. | What degree have you obtained as a qualified speech therapist? (1) Bachelor’s degree or (2) Master’s degree. |
| 2. | Where have you been trained as a qualified speech therapist? (1) Hong Kong or (2) English-speaking country (e.g., the US, the US, etc.). |
| 3. | Are you a Cantonese speaker? (1) Yes or (2) No |
| 4. | What other languages do you speak? (Can select more than one answer) (1) English, (2) Mandarin, or (3) Others (mandatory text input required). |
| 5. | What language(s) do you speak for providing professional speech therapy services? (Can select more than one answer) (1) Cantonese, (2) English, (3) Mandarin, or (4) Others (mandatory text input required). |
| 6. | Do you have any post-qualification degrees? (1) Research master’s degree (e.g., MPhil), (2) Research doctoral degree (e.g., PhD), (3) Degree from a taught postgraduate program (master’s level), (4) Degree from a taught postgraduate program (doctoral level), or (5) None. |
| 7. | What setting(s) are you currently working in? (Can select more than one answer) (1) NGOs [e.g., EETC, ICCC, SCCC, OPRS, or others], (2) Special school, (3) Mainstream school, (4) Private clinic, (5) Hospital, (6) University, (7) Government (e.g., Department of Health, EDB, or others), or (8) Others (mandatory text input required). |
| 8. | How many years and months of clinical experience do you have? Please indicate as years and months, e.g., 10 years 10 months. |
| 9. | How would you describe your understanding of CAS in Cantonese speakers? (1) Very little, (2) A little, (3) Fair, (4) A great deal, or (5) A very great deal. |
| 10. | How did you obtain training about CAS? (Can select more than one answer) (1) I did not have any training about CAS, (2) Professional degree (e.g., Bachelor’s or Master’s degree), (3) Continuing education program before or after graduating as a qualified speech therapist, (4) Self-instruction (e.g., online journal articles or resources), or (5) Others (mandatory text input required). |
| 11. | Follow up question 1/4: Please indicate how much (in %) your training from (1) professional degree (i.e., Bachelor’s or Master’s degree) contributed to your understanding of CAS. (Text input required.) |
| 12. | Follow up question 2/4: Please indicate how much (in %) your training from (2) continuing education program(s) before or after graduating as a qualified speech therapist contributed to your understanding of CAS. Please provide the name(s) or specific topics of the courses you took. (Text input required.) |
| 13. | Follow up question 3/4: Please indicate how much (in %) your training from (3) self-learning (e.g., online journal articles or resources) contributed to your understanding of CAS. Please provide examples. (Text input required.) |
| 14. | Follow up question 4/4: Please indicate how much (in %) your training from (4) others contributed to your understanding of CAS. (Text input required.) |
| 15. | How many Cantonese-speaking children have you worked with who were diagnosed with/suspected to have CAS? (1) 0, (2) 1-5, (3) 6-10, (4) 11-15, (5) 16-20, or (6) >20. |
| 16. | With respect to the children in the last question, how many of them were initially diagnosed/suspected by you? (1) 0, (2) 1-5, (3) 6-10, (4) 11-15, (5) 16-20, or (6) >20. |
| 17. | What is the percentage of Cantonese-speaking children with CAS or suspected CAS on your current caseload? (1) 0%, (2) 1%-20%, (3) 21%-40%, (4) 41%-60%, (5) 51%-80%, or (6) 81%-100%. |
| Assessment and diagnosis | |
| 18. | What assessment tasks do you use to assist in the process of making a differential diagnosis of CAS in Cantonese speakers? (1) Case history, (2) Speech and language sample, (3) Nonspeech oral motor examination, (4) Standardized or nonstandardized language assessment, (5) Standardized articulation or speech production test (e.g., HKCAT or CSPT), (6) Nonstandardized speech assessment tasks, (7) Imitation and/or production of polysyllabic words, (8) Diadochokinetic [DDK] tasks, including AMR and SMR, (9) Production of words of increasing length, (10) Observation of prosody, (11) Speech perception tasks/tests, or (12) Others (mandatory text input required). |
| 19. | Do you use standardized assessment tools to diagnose Cantonese speakers with CAS? If yes, please specify. (Text input required.) |
| 20. | Do you use objective measures/tasks to assist in your diagnostic process? If yes, please specify which task(s) you use. (Text input required.) |
| 21. | Do you use any available checklist(s) of clinical features to diagnose Cantonese speakers with CAS? If yes, please write down the name and reference of the checklist. (Text input required.) |
| 22. | Do you use your own set of clinical features to diagnose Cantonese-speaking children with CAS? If yes, please list them all. (Text input required.) |
| 23. | A checklist is used with a set of criteria, for example, "4 out of 10 features observed from 3 different tasks" is used with Strand's 10-point checklist. Referring to the last question, please list your set of criteria for the features you use for making a differential diagnosis of CAS in Cantonese speakers. (Text input required.) |
| 24. | How do you rate the importance of "inconsistent errors" in making a differential diagnosis of CAS in Cantonese speakers? 7-point scale: 1 = not important at all; 7 = very important. |
| 25. | How do you rate the importance of "difficulty in movement transition between syllables or syllable segregation" in making a differential diagnosis of CAS in Cantonese speakers? 7-point scale: 1 = not important at all; 7 = very important. |
| 26. | How do you rate the importance of "lexical tone errors" in making a differential diagnosis of CAS in Cantonese speakers? 7-point scale: 1 = not important at all; 7 = very important. |
| 27. | How do you rate the importance of "poor DDK performance" in making a differential diagnosis of CAS in Cantonese speakers? 7-point scale: 1 = not important at all; 7 = very important. |
| 28. | How do you rate the importance of "poor performance on polysyllabic words" in making a differential diagnosis of CAS in Cantonese speakers? 7-point scale: 1 = not important at all; 7 = very important. |
| 29. | How do you rate the importance of "nonspeech groping behaviours" in making a differential diagnosis of CAS in Cantonese speakers? 7-point scale: 1 = not important at all; 7 = very important. |
| 30. | How do you rate the importance of "within-speech groping behaviours" in making a differential diagnosis of CAS in Cantonese speakers? 7-point scale: 1 = not important at all; 7 = very important. |
| Treatment | |
| 31. | How frequently (per week) do you provide treatment to children with CAS? (1) Less than once a week, (2) Once a week, (3) Twice a week, (4) Three times a week, (4) Four times a week, or (5) More than four times a week. |
| 32. | How much time (in minutes) does each treatment session last? (1) <30, (2) 30-34, (3) 35-50, (4) 51-60, (5) >60, or (6) Others (mandatory text input required). |
| 33. | How do you organize the treatment sessions with respect to speech production practice versus other goals? (1) Within-session block treatment [i.e., include speech production practice and other targets in the same session] or (2) Between-session block treatment [i.e., several sessions for speech production and several sessions for other targets]. |
| 34. | If you are using "within-session block treatment", what is the estimated percentage of speech production practice in the sessions youprovide to children with CAS? (1) 10%, (2) 20%, (3) 30%, (4) 40%, (5) 50%, 6) 60%, (7) 70%, (8) 80%, or (9) 90%. |
| 35. | What is/are the other area(s) that you target for children with CAS? (1) Speech perception skills, (2) Language skills, (3) Nonspeech oral motor abilities, (4) Literacy skills, (5) Social skills, or (6) Others (Mandatory text input required). |
| 36. | If you are using "between-session block treatment", how many sessions are in the block of speech production practice? (If not applicable, please type “0”.) (Text input required.) |
| 37. | If you are using "between-session block treatment", how many sessions are in the block of other targets? (If not applicable, please type “0”.) (Text input required.) |
| 38. | In speech production treatment, which area do you usually work on first for children with CAS? (1) Segments (e.g., phoneme and syllable structure accuracy), (2) Suprasegments (e.g., co-articulation, speech rate, lexical tones, etc.), or (3) Both segments and suprasegments. |
| 39. | What is/are the reason(s) for the choices you indicated in the previous question? (Text input required.) |
| 40. | In speech production treatment, please indicate the priority of treatment for the following types of segments. Checkbox Grid row: (1) Initial consonants, (2) Vowels/diphthongs, (3) Final consonants, and (4) Syllable structures; Column (1) 1^st^, (2) 2^nd^, (3) 3^rd^, and (4) 4^th^. |
| 41. | What is/are the reason(s) for the choices you indicated in the previous question? (Text input required.) |
| 42. | In speech production treatment, please indicate the priority of treatment for the following types of suprasegmental features. Checkbox Grid row: (1) Speech rate, (2) Intonation, (3) Co-articulation, and (4) Lexical tones; Column (1) 1^st^, (2) 2^nd^, (3) 3^rd^, and (4) 4^th^. |
| 43. | What is/are the reason(s) for the priorities you indicated in the previous question? (Text input required.) |
| 44. | Which of the following evidence-based treatment approaches do you use for children with CAS? (1) Dynamic Temporal and Tactile Cueing, (2) Rapid Syllable Transition (ReST), (3) Nuffield Dyspraxia Program, (4) Ultrasound biofeedback, (5) Integrated Phonological Awareness, (6) PROMPTS for Restructuring Oral Muscular Phonetic Targets (PROMPT), (7) Augmentative and Alternative Communication [AAC], or (8) None. |
| 45. | Please leave this question blank if you selected "none" in the last question. How much of the evidence-based treatment program(s) that you selected above have you implemented when you have treated a child with CAS? 7-point scale: 1 = Minimal implementation; 7 = Full implementation. |
| 46. | What is your reason for your full/partial implementation of the treatment program(s) indicated above? (Text input required.) |
| 47. | Have you used any of the following treatment approaches for children with CAS in your practice? If any approach you use is not listed, please describe in as much detail as possible in "others". (1) Kaufman Apraxia Treatment, (2) Melodic Intonation Therapy, (3) Talktools® Oral Placement Therapy, (4) Syllable Repetition Method (e.g., production of [papapa], [papapi], [papipu], etc.), (5) Speech Motor Learning (SML) approach, (6) Traditional articulation approach, (7) Minimal Pair approach, or (8) None. |
| 48. | Why did you choose the treatment approach selected above for children with CAS? (1) It is an evidence-based approach, (2) I am familiar with this approach, (3) the treatment is easy to implement, (4) the treatment matches with the clients’ deficits/characteristics, or (5) Others (mandatory text input required). |
| *Abbreviations.* AMR = alternate motion rate; CAS = childhood apraxia of speech; CSPT = Cantonese Segmental Phonology Test [1]; DDK = diadochokinesis; EDB = Education Bureau; EETC = Early education and training center; HKCAT = Hong Kong Cantonese Articulation Test [2]; ICCC = Integrated program in kindergarten-cum child care center; NGOs = nongovernment organizations; OPRS = On-site preschool rehabilitation services; SCCC= special child care center; SMR = sequential motion rate | |

**References**

1. So LKH. Cantonese segmental phonology test. Hong Kong: Bradford Publishing Company; 1993.

2. Cheung PSP, Ng KH, To C. Hong Kong Cantonese Articulation Test. Hong Kong: Language Information Sciences Research Centre and The City University of Hong Kong; 2006.
